# Comprehensive analysis of ectopic mandibular third molar: a rare clinical entity revisited

**DOI:** 10.1186/s13005-017-0157-x

**Published:** 2017-12-11

**Authors:** Yaping Wu, Yue Song, Rong Huang, Jiaan Hu, Xiaotong He, Yanling Wang, Guangchao Zhou, Chao Sun, Hongbing Jiang, Jie Cheng, Dongmiao Wang

**Affiliations:** 1 Department of Oral and Maxillofacial Surgery, Affiliated Hospital of Stomatology, Nanjing Medical University, Nanjing, 210029 China; 2 Jiangsu Key Laboratory of Oral Disease, Nanjing Medical University, Nanjing, 210029 China; 3 Department of Oral and Maxillofacial Radiology, Affiliated Hospital of Stomatology, Nanjing Medical University, Nanjing, 210029 China

**Keywords:** Ectopic tooth, Mandibular third molar, Impacted third molar, Panoramic radiography

## Abstract

**Background:**

Ectopic mandibular third molar is a rare clinical entity with incompletely known etiology. Here, we sought to delineate its epidemiological, clinical and radiographic characteristics, and therapy by integrating and analyzing the cases treated in our institution together with previously reported cases.

**Method:**

A new definition and classification for ectopic mandibular third molar was proposed based on its anatomic location on panoramic images. Thirty-eight ectopic mandibular third molars in 37 patients and 51 teeth in 49 patients were identified in our disease registry and from literature (1990–2016), respectively. These cases were further categorized and compared according to our classification protocol. The demographic, clinicopathological and radiographic data were collected and analyzed.

**Results:**

These ectopic teeth were categorized into four levels, 33 in level I(upper ramus), 32 in level II (middle ramus), 15 in level III (mandibular angle) and 9 in level IV (mandibular body). The common clinical presentations included pain, swelling and limited mouth opening, although sometimes asymptomatic. Most teeth were associated with pathological lesions. Treatments included clinical monitor and surgical removal by intra- or extraoral approach with favorable outcomes. Clinical presentations and treatment options for these teeth were significantly associated with their ectopic locations as we classified.

**Conclusions:**

Ectopic mandibular third molars are usually found in patients with middle ages and in upper and middle ramus of mandible. Surgery is preferred to remove these ectopic teeth and associated pathologies when possible.

## Background

Ectopic tooth is usually diagnosed when the tooth is displaced in an unusual location and with a distance from its normal anatomic site. It may be supernumerary, deciduous, or permanent tooth which has been reported in diverse heterotopic positions including nasal cavity, maxillary sinus, orbit, palate, mandibular condyle and coronoid process [1–5]. Noticeably, given that mandibular third molar is the most frequently impact tooth with a prevalence of approximately 20–30%, limited eruption space or barriers in its eruption trajectory tends to make the lower third molar displaced away from its physiologic eruption region [6–8]. However, ectopic mandibular third molar is relatively rare, since only a few cases have been reported in the literature [1, 3, 9–18]. Owing to the rarity of this condition, the etiology, clinical manifestations and optimal management of ectopic mandibular third molar are still not well-established until now.

The etiologic cause for ectopic tooth remains unknown thus far. It might be resulted from developmental disturbances of jaws or pathological process or an iatrogenic activity. Several ideas have been proposed to explain ectopic displacement of mandibular third molar such as aberrant eruption, trauma, and ectopic formation of tooth germ [19]. Previous reports have been documented various non-physiologic sites for these displaced molars such as the board or angle of mandible, the ramus, condyle, coronoid process, or even in the adjacent soft tissue spaces [1–3, 7, 9, 20]. Most ectopic mandibular third molars are found to be associated with pathological lesions including odontogenic cysts or tumors [3, 8, 21–23]. Some ectopic third molars are asymptomatic, sometimes over the period of lifetime and are usually incidentally found during routine clinical and radiographic investigations [24]. Pain, swelling and limited mouth opening/trismus are the most common clinical features, especially when these teeth are associated with certain pathological lesions or the surrounding tissue is affected [8, 9, 13]. Their diagnose are largely based on the radiographic findings from panoramic radiography or computed tomography (CT) [10]. To determine the precise locations for these ectopic mandibular third molars, various diagnostic images were used. Panoramic radiograph is generally sufficient for making the diagnosis of an ectopic third molar [1, 18]. Three-dimensional anatomical position of ectopic mandibular third molar and its relation to surrounding anatomical structures can be evaluated with CT or dental come-beam CT (CBCT) [10]. However, in these previous case reports, the detailed locations of ectopic mandibular third molars were mapped in a descriptive manner and varied significantly among diverse studies. To the best of our knowledge, no unified classification for ectopic sites of mandibular third molar has been reported, thus hindering the comprehensive analyses of these rare entities. There is only one report which has documented three types of tooth displacements of the impacted mandibular third molars caused by cystic lesions. However, such displacements can’t always be defined as ectopic but might be regarded as impaction [25].

Therapeutic options for the ectopic mandibular third molar are based on the clinical presentations, surgical risks and complications as well as patient preference [3, 8]. If the tooth is asymptomatic, it can be closely monitored with regular follow-up. Surgical intervention is indicated when the tooth is associated with pathological lesion or it cause discomfort and clinical symptoms. The intraoral approach is preferred as a routine technique and considered to be more conservative, although it has the limitation of inadequate visualization and lack of surgical fields [2, 10, 26]. By contrast, the extraoral approach is selected to get access to the tooth directly but with the possibility to damage the facial nerve and leave facial cutaneous scar [11, 12].

The aim of the present study was to report the ectopic mandibular third molars treated at our institution and introduce a new classification for these ectopic teeth based on their anatomic locations on panoramic images. Furthermore, the previously published cases together with our patients were combined and classified in terms of our classification regime to comprehensively analyze this rare clinical entity.

## Methods

### Definition and classifications of ectopic mandibular third molar based on panoramic radiographic findings

**Fig. 1:**
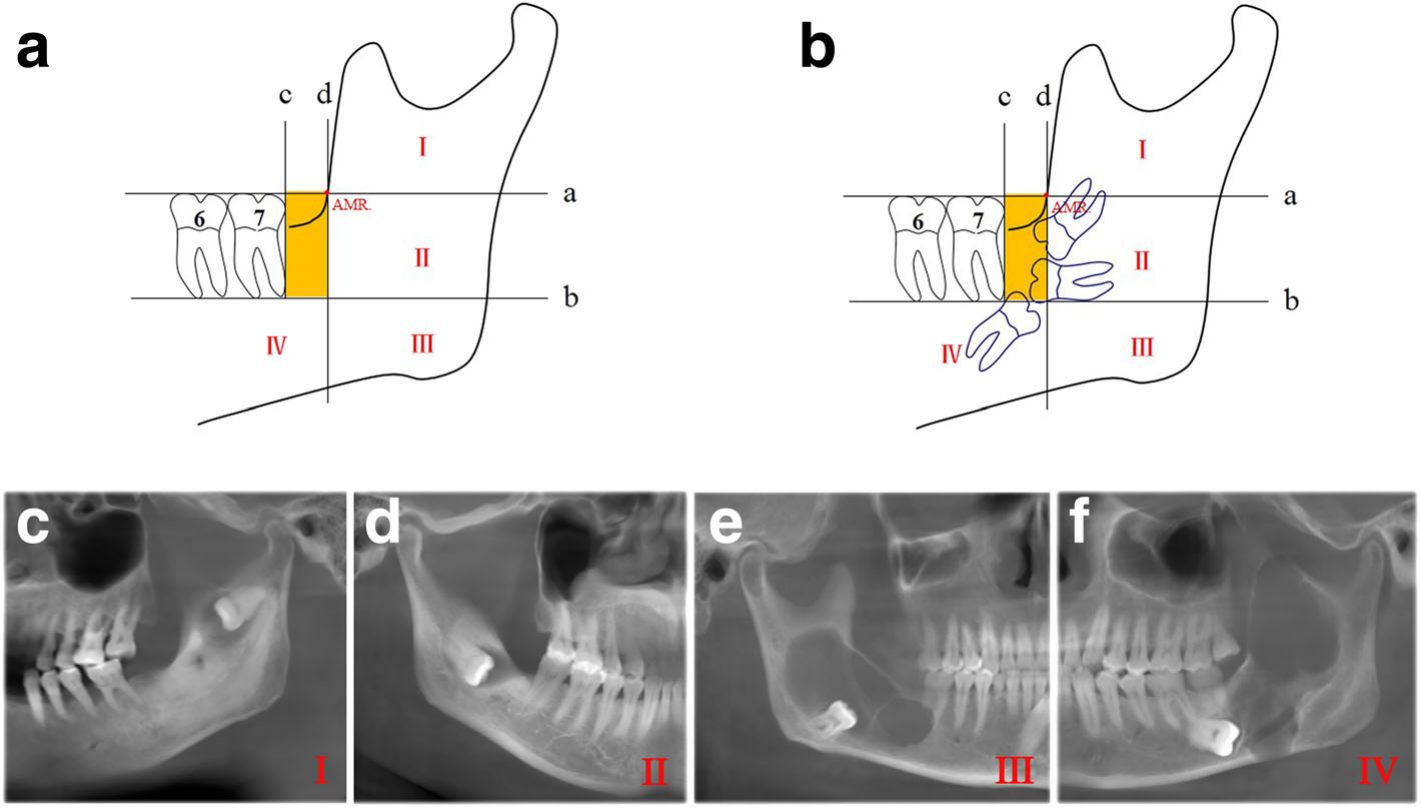
Definition and classification of ectopic mandibular third molar based on panoramic images. **a:** diagram describing the definition and classification of ectopic mandibular third molar based on panoramic images. The line a was drawn from the occlusal plane of the mesial teeth such as the second and/or first molar. The intersection point between the line a and anterior mandibular ramus was defined as AMR (anterior mandibular ramus). The line b was extended from the root tips of the second molar or first molar when second molar was absent, which was also in parallel to the line a. The line c was perpendicular to line a and tangential through the distant contour of the mesial tooth. The line d was through the AMR point and parallel to the line c. Thus, the area surrounded by the four lines was defined as the physiological region (marked as the yellow area) for the third molar eruption. Therefore, four levels (I-IV) were defined as ectopic locations for the mandibular third molar. **b:** Any parts of the third molar which fall into the putative normal region as marked as the yellow area or contact with this area were defined as impaction rather than ectopic and excluded here. **c-f:** representative images of level I-IV ectopic third molar in mandible

Given the paucity of the unified classification for ectopic mandibular third molar, we reviewed the radiographic findings of patients who were previously diagnosed as ectopic mandibular third molars in literature. We proposed a new definition and classification for ectopic mandibular third molar based on its anatomic site on the routine panoramic images either derived from CBCT or panoramic radiograph. On the panoramic radiograph or the panoramic images derived from original CBCT, as shown in Fig. 1a, the line a was drawn from the occlusal plane of the mesial teeth such as the second and/or first molar. The intersection point between the line a and anterior mandibular ramus was defined as AMR (anterior mandibular ramus). The line b was extended from the root tips of the second molar or first molar when second molar was absent, which was also in parallel to the line a. The line c was perpendicular to line a and tangential through the distant contour of the second molar. The line d was through the AMR point and in parallel to the line c. Thus, the area surrounded by the four lines was defined as the physiological region for the third molar eruption. Accordingly, the mandible involved the ectopic third molar were further divided into four levels (I-IV). As indicated in Fig. 1 b, we adopt a more stringent definition of ectopic third molar as when any parts of the third molar fall into or contact with the putative physiologic eruption region as marked by yellow area, the tooth was defined as impaction rather than ectopic and thus excluded from the present study. The representative images of the four classification for ectopic third molars from our patient cohort were further shown in Fig. 1c-f.

### Patient screen and inclusion in our patient registry

**Fig. 2:**
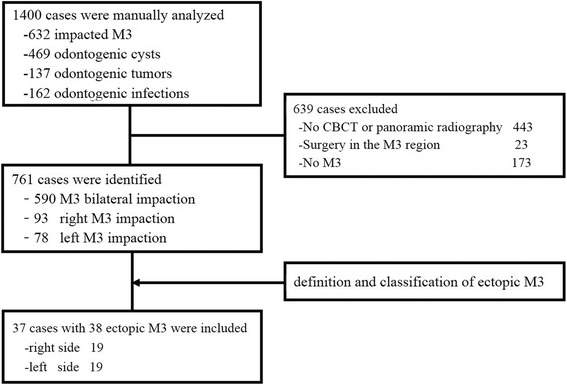
Patients screen and inclusion protocol

The oral and maxillofacial disease registry at our institution was electronically searched to identify patients who were previously diagnosed as impacted mandibular third molar, mandibular odontogenic cysts or tumors and odontogenic infections between January 2012 and December 2016. The medical chart, radiographic and relevant pathological data for each candidate were manually reviewed to confirm these patients with ectopic mandibular third molars. The detailed inclusion criteria included patients with ectopic mandibular third molars which were definitively diagnosed based on our proposed radiographic definition/classification and complete information about patients’ demographics, associated clinicopathological features, treatment modalities as well as outcomes. Moreover, the patient exclusion criteria were listed as follows: 1) no mandibular third molars; 2) tooth with not completely developed root; 3) previous surgical procedures involved in this region, for example decompression or marsupialization for cystic lesions; 4) historical or ongoing orthodontic treatments; 5) the poor quality of CBCT or panoramic images jeopardizing unambiguous view of local anatomy and structures. The detailed screen and inclusion/exclusion of patients were described in Fig. 2. The research protocol (2016–256) was reviewed and approved by the Ethics and Research Committee of Nanjing Medical University. Written informed consent was obtained from all enrolled patients before treatment. The methods and protocols during the whole study were performed in accordance with the tenets of the Declaration of Helsinki for research involving human subjects as well as our institutional guidelines.

To verify the diagnosis of ectopic mandibular third molar, the qualified panoramic images were analyzed by two trained observers with high expertise (Dr. Yaping Wu and Dr. Rong Huang). When different ideas concerning the radiographic findings occur, they should discuss with another independent observer (Dr. Yanling Wang) and then obtain the final consensus. To testify the reliability and reproducibility of our definition and classification for ectopic mandibular third molar, these measurements for our patients were initially performed independently by two observers or repeatedly performed by one observer (Dr. Yaping Wu) at two time points with one month interval, which were further statistically compared.

### Literature search strategy and patient inclusion

**Fig. 3:**
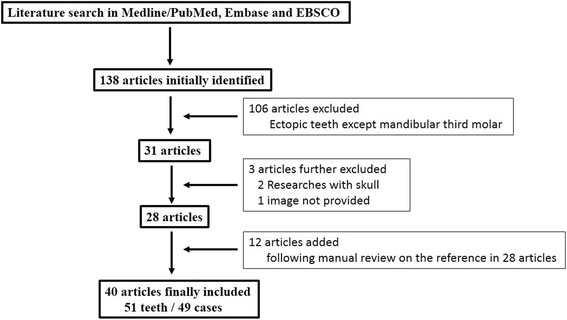
Protocol of literature review and case selection

We conducted a comprehensive literature search in electronic databases including Medline/PubMed, Embase and EBSCO using the keywords “ectopic/displaced tooth”, “ectopic/displaced molar”, “impacted lower third molar” and “impacted mandibular third molar”. This literature search was confined to English language and human studies. Here we chose 1990 as the initial year and 2016 as terminal year largely due to the availability of qualified images in the literature. As described in Fig. 3, the abstracts, titles or full text of results were reviewed to eliminate the obvious unrelated articles and include those with great relevance. A manual search of the reference lists of these selected articles was also conducted to find additional articles that might fit the selection criteria. The inclusion criteria for patients from the literature were the same as patient selection in our cohort. Studies from cadavers and iatrogenic displacement of third molar were excluded. All relevant clinical, pathological as well as radiographic information regarding patients’ demographic, sides and locations of ectopic teeth, clinical symptoms and pathological diagnosis and treatment modalities were extracted from the included articles. These included cases were further categorized based on our proposed classification and analyzed similarly as our patients.

### Statistical analysis

All relevant data including the demographic, clinical and radiographic data of patients were collected. Associations between categorical covariates were assessed by Fisher exact test as indicated. The intra-observer and inter-observer agreements of these descriptive radiographic findings in our patient cohort were estimated by Cohen κ test. Statistical analyses were performed using Stata 9.2 software program or GraphPad Prism 7.0with indicated methods. *P* < 0.05 (two-sides) was considered statistically significant.

## Results

### Clinicopathological characteristics of ectopic mandibular third molar in our patient cohort

Through patient screen in our registry system, we identified 37 patients (38 teeth in total) satisfying the inclusion criteria between 2012 and 2016. As listed in Table 1, the mean age was 36.30 ± 13.48 years (range:18–69 years), with 22 males and 15 females. Thirty-six cases were presented as unilateral (18 in the right and 18 in the left), and one case was bilateral. Moreover, based on the our classification, these ectopic mandibular third molars were categorized into level I (2), level II (21), level III (8), level IV (6), respectively. Good intra-observer and inter-observer reliabilities (κ = 0.93, *P* = 0.002; κ = 0.87, *P* = 0.013, respectively) as estimated by the κ-test analyses were found to support the notion that our definition and classification for ectopic mandibular third molar were reproducible and reliable. The most frequent clinical presentations were pain and/or swelling on the ipsilateral side of the mandible, limited mouth opening/trismus, as well as combinations of these symptoms or no discomfort. Five patients suffered with pain, swelling and trismus, 14 patients with pain and swelling, 6 with swelling, and 5 with pain only. Eight patients appeared free of symptoms and incidentally identified by panoramic radiograph. As shown in Table 2, we found that the occurrence of swelling was significantly associated with the diverse regions of ectopic teeth (*P* < 0.033, Fisher exact test). In addition, limited mouth opening/Trismus was also tend to associated with different ectopic locations, although the difference didn’t reach statistical significant (*P* = 0.078, Fisher exact test). Most patients (36) were found to be associated with pathological lesions, including dentigerous cyst, odontogenic keratocyst, ameloblastoma, myxoma and inflamed granulation tissue. All teeth received the surgical removal intra-orally (37) and extra-orally (1) under general anesthesia. These associated pathological lesions were also simultaneously removed and histopathologically diagnosed. Most patients (32/37) had uneventful recovery. Five patients (5/37) had short-term swelling, limited mouth opening as well as temporal neurosensory disturbance resulted from inferior alveolar nerve damage and recovered weeks later. However, no significant association was detected between treatment approaches and ectopic locations (Table 3).

**Table 1:** Descriptive data of 38 ectopic mandibular third molars in 37 patients

Variable		No. (%)
Age (years)	36.30 ± 13.48(18–69)	
Gender	Male	22 (59.46%)
	Female	15 (40.54%)
Ectopic side	Right side	18 (48.65%)
	Left side	18 (48.65%)
	Bilateral sides	1 (2.70%)
Ectopic type	Level I	3 (7.89%)
	Level II	21 (55.26%)
	Level III	8 (21.05%)
	Level IV	6 (15.79%)
Symptom/sign	Present	30 (78.95%)
	Absent	8 (21.05%)
Associated pathology	Free	2 (5.26%)
	Odontogenic/dentigerous cyst	15 (39.47%)
	Odontogenic tumor	20 (52.63%)
	inflammation	1 (2.63%)
Surgical approach	Intra-oral	36 (94.74%)
	Extra-oral	1 (2.63%)
	No treatment	1 (2.63%)

**Table 2:** Clinical presentations in 37 patients with 38 ectopic mandibular third molars

Symptoms	Swelling		*P*	Pain		*P*	Limited mouth opening/Trismus		*P*
	Yes	No		Yes	No		Yes	No	
**Level I**	3	0	**0.033**	2	1	0.907	2	1	0.078
**Level II**	11	10		12	9		2	19	
**Level III**	8	0		6	2		1	7	
**Level IV**	3	3		4	2		0	6	

Note: The *P*-value is calculated by Fisher exact test using GraphPad Prism 7.0. The numbers in bold indicate statistical significant

**Table 3:** Treatment approaches for 37 patients with 38 ectopic impacted mandibular third molars

	Surgical approach			*P*
	Intra-oral	Extra-oral	No treatment	
Level I	2	1	0	0.283
Level II	20	0	1	
Level III	8	0	0	
Level IV	6	0	0	

Note: The *P*-value is calculated by Fisher exact test using GraphPad Prism 7.0

### Comprehensive review of 89 ectopic mandibular third molar from literature and our patient cohort

As shown in Fig. 3, comprehensive literature reviews have identified a total number of 49 published cases with 51 ectopic mandibular third molars in literature from 1990 to 2016 [2, 3, 7–21, 24, 26–46]. These cases and 37 unpublished cases presented here were combined and further analyzed with respect to diverse clinicopathological variables. The epidemiological and clinical characteristics of these cases are summarized in Table. 4. Among the 86 patients reported in the present study, the mean age was 41.72 ± 14.14 years (range:18–80 years), with 40 males and 46 females. Eighty-three cases were unilateral (43 in the right and 40 in the left side) and three cases were bilateral. Based on the original images and relevant description in the literature, these ectopic third molars were located in different regions of mandible, including condyle, subcondyle, sigmoid notch, coronoid process, ascending ramus, angle of the mandible and the boarder of the mandibular body. As listed in Table 4, the most common region for ectopic mandibular third molar is level I (33), followed by level II (32), level III (15) and level IV (9). The common presentations included pain and swelling on ipsilateral side of the mandible or the preauricular region, trismus, difficulty chewing, and cutaneous fistula, etc. Most cases were associated with pathological lesions, including odontogenic cysts or tumors, while 10 case were free of associated pathologies. With regard to the treatment approach, most teeth as well as associated pathological lesions were surgically removed by intraoral or extraoral approaches. Four patients were not operated and monitored frequently due to diverse reasons such as risk of condyle fracture/loss, no discomfort as well as patient preference. Interestingly, as shown in Table 5, the clinical presentations such as swelling and limited mouth opening was found to be significantly associated with diverse ectopic locations of ectopic teeth (*P* = 0.002, 0.021, respectively; Fisher exact test). Moreover, different treatment approaches were also found to be also significantly associated with locations of these ectopic teeth as we classified (*P* = 0.001, Fisher exact test, Table 6).

**Table 4:** Descriptive data about 86 patients with 89 ectopic mandibular third molars in our cohort and English literature (1990–2016)

Variable		No. (%)
Age (years)	41.72 ± 14.14(18–80)	
Gender	Male	40 (46.51%)
	Female	46 (53.49%)
Ectopic side	Right side	43 (50.00%)
	Left side	40 (46.51%)
	Bilateral sides	3 (3.49%)
Ectopic type	Level I	33 (37.08%)
	Level II	32 (35.96%)
	Level III	15 (16.85%)
	Level IV	9 (10.11%)
Symptom/sign	Present	77 (86.52%)
	Absent	12 (13.48%)
Associated pathology	Free	10 (11.24%)
	Odontogenic/dentigerous cyst	47 (52.81%)
	Odontogenic tumor	20 (22.47%)
	Inflammation	8 (8.99%)
	Not mentioned	4 (4.49%)
Surgical approach	Intra-oral	65 (73.03%)
	Extra-oral	20 (22.47%)
	No treatment	4 (4.49%)

**Table 5:** Clinical presentations in patients with ectopic mandibular third molars

Symptoms	Swelling		*P*	Pain		*P*	Limited mouth opening		*P*	Fistula		*P*
	Yes	No		Yes	No		Yes	No		Yes	No	
Level I	26	7	**0.002**	24	9	0.730	13	20	**0.021**	3	30	0.666
Level II	17	15		21	11		3	29		1	31	
Level III	15	0		11	4		2	13		1	14	
Level IV	5	4		5	4		1	8		1	8	

Note: The *P*-value is calculated by Fisher exact test using GraphPad Prism 7.0. The numbers in bold indicate statistical significant

**Table 6:** Treatment approaches for patients with ectopic mandibular third molars

	Surgical approach			*P*
	Intra-oral	Extra-oral	No treatment	
Level I	19	12	2	**0.001**
Level II	30	0	2	
Level III	10	5	0	
Level IV	6	3	0	

Note: The *P*-value is calculated by Fisher exact test using GraphPad Prism 7.0. The numbers in bold indicate statistical significant

## Discussion

Ectopic mandibular third molar is rare in the dental clinic and usually found at heterotopic places distant from their physiological sites of eruption [2, 3, 8]. Until now, a few case reports or small case series have been reported so far. However, the accurate definition and unified classification for these ectopic teeth is still lacking, which profoundly complicates the clear understanding about its etiology, clinical features as well as optimal therapy. Here we proposed a stringent definition and new classification of ectopic mandibular third molar based on panoramic images and presented the epidemiological, clinical features and treatment of this rare entity by integrating the data from our patient cohort and literature. To the best of our knowledge, this might be a clinical study concerning ectopic mandibular third molar with the largest number of patients which offers comprehensive clinical, pathologic and radiographic information.

### Etiology, definition and classification for ectopic mandibular third molar

The etiology of ectopic mandibular third molars has not yet been completely clarified until now. Some hypotheses have been proposed to explain these rare conditions, for example, trauma, deviant position of tooth gem, aberrant eruption pattern or displaced by pathological lesions such as cyst or tumor in the jaw [3, 8]. Notably, the associated lesions such dentigerous cyst might be the most frequent cause of the dislocation, which was supported by most reports and our results [7, 8]. Most of the reported cases were found associated with radiolucent lesions in the panoramic radiograph and confirmed histopathologically as cysts or tumors. In the present patient cohort and the cases mined from literature, the overweighing majority of ectopic teeth is found simultaneously with odontogenic cysts or tumors [40]. The pressure resulted from these associated lesions displaces tooth buds in an abnormal direction which were further developed in the ectopic region [25, 37]. Moreover, local inflammations support the progressive expansion of cysts and serves as an additional factor to force the migration of the tooth into an ectopic position [17]. However, some cases are not involved with any pathological lesions, thus other etiological factors in its pathogenesis can’t be ruled out [9, 31, 39].

As to the definition of ectopic tooth, it is usually defined as the tooth located in a non-physiologic area. The common unerupted/impacted mandibular third molar may confound the diagnosis of ectopic third molar largely due to the lack of accurate and unified definition of this rare condition. The definition used in previous studies varied individually. To address this issue, we propose a new stringent definition and classification regime about the ectopic mandibular third molar. Our definition can easily differentiate the ectopic third molars from those impacted/unerupted third molars. Moreover, our data revealed good reliability and reproducibility of this regime and further found that diverse subtypes of ectopic teeth significantly associated with different clinical presentations as well as therapeutic approaches, thus initially demonstrating its utility in clinical diagnosis and treatment for ectopic mandibular third molar. Collectively, our definition and classification might be a new start point to optimize the clinical diagnosis and treatment for ectopic mandibular third molar.

### Patient demographics and epidemiological characteristics of ectopic mandibular third molar

The patients with ectopic mandibular third molars were diagnosed at mean age 41.72 years, with a slight female predilection, which is generally with previous reports [2, 31]. This epidemiological features are different from the impacted teeth which are commonly detected in much younger ages. We believe that ectopic mandibular third molars are usually asymptomatic and incidentally identified unless prominent discomfort or clinical presentations occur, which might account for the late age of diagnosis for ectopic teeth. Most ectopic teeth were found in one side of mandible, and extremely rare in both sides [30]. Interestingly, most ectopic teeth were identified in levelI and II as defined in our classification regime, followed by mandibular angle and lower border (level III and IV), which is well consistent with previous reported frequent locations of ectopic third molar such as condyle, subcondyle and sigmoid notch [1, 3, 10, 14, 18, 30] and rare sites such as mandibular lower border [12].

### Clinical features and associated pathologies of ectopic mandibular third molar

The ectopic mandibular third molar is usually insidious, asymptomatic and difficult to identify in advance [2]. The common clinical presentations include pain, swelling, limited mouth opening, discharging fistula or TMJ discomfort [2, 3, 8]. However, these are non-specific and resulted from the surrounding tissue affected. The diagnosis of ectopic mandibular third molar relies heavily on radiographic findings such panoramic radiograph or CT/CBCT. CT images can offer more accurate 3-dimensional information regarding the precise location of the ectopic tooth and the surrounding structures [10, 32]. Intriguingly, our data further revealed that ectopic locations as we classified was found to be significantly associated with symptoms such as swelling and limited mouth opening which were presumably caused by resultant inflammation in surround tissue triggered by displaced teeth.

Consistent with previous reports, most ectopic mandibular third molars were found simultaneously with pathological lesions like odontogenic cysts or tumors [2, 27, 40]. On the panoramic images, the tooth crown was typically surrounded by radiolucent lesions (odontogenic cysts) or the tooth was embedded into the lesion [29, 47]. In some occasions, clinical symptoms and discomfort might be resulted from these associated pathologic lesions. Our results and previous literature support the hypothesis that these associated lesions might etiologically contribute to ectopic displacement of mandibular third molar [3, 8, 9].

### Treatment strategy and outcomes of ectopic mandibular third molar

Clinical management of ectopic mandibular third molar depends on several factors such as symptoms, associated pathology, surgical risk and complications as well as patient preference [2, 7, 8]. When the tooth is asymptomatic and without associated lesions, treatment can be conservative and patients are monitored with follow-up at regular intervals. These ectopic teeth should be treated if they cause symptoms, associate with inflammation, cystic disease or odontogenic tumors, and to prevent future complications. Careful treatment planning should be made and the selection of surgical approach is basically linked to the location of tooth, experience of surgeons, and surgical morbidity. Most patients can be treated in an intraoral approach whenever possible, sometimes with assistance of endoscope, to avoid visible facial scar and facial nerve damage [44]. However, in some instances, intraoral approach is not appropriate as it can’t provide adequate surgical field and clear visualization in these inaccessible anatomical area or in patients with severe restriction of mouth opening [11, 43]. Our findings indicate that the ectopic teeth located in level III and IV tend to be extracted in an extraoral approach by submandibular or retromandibular incision to get good surgical exposure. Particularly, when the ectopic tooth was located in the condyle, the preauricular or transmasseteric antero-parotid approach can be utilized to reduce surgical morbidity and the risk of iatrogenic injury [11]. Careful attention should be paid to the critical situation when the ectopic tooth is displaced at the condyle or condyle neck, because the remaining bone is thin and vulnerable to pathological fracture or resorption [7, 16, 26, 41]. Moreover, the risk of neuronal structures damage, for example when the tooth has close proximity with the inferior alveolar nerve, should also be evaluated before treatment. The utilization of CBCT or high-resolution CT scans can provide accurate information about the detailed 3-dimensional location of these ectopic teeth as well as the topographic relationship between the teeth with surrounding vital structures, which is highly beneficial to select appropriate surgical route and strategy [10, 32, 48]. It’s clear that the ectopic mandibular third molar and the accompanying pathological lesions are usually indicated for simultaneous removal if possible. In some occasions when the cystic lesions occupy the most parts of mandibular ramus, the teeth might be extracted in second stage following cyst decompression or marsupialization.

### Advantages and limitations of definition and classification of ectopic mandibular third molar

Our definition and classification of ectopic mandibular third molar might have advantages as well as shortcomings. This definition and classification regime is relatively simple, reproducible and easily to communicate among clinicians. Moreover, this regime might be valuable to understand the epidemiological, clinical and radiographic characteristics of this rare clinical entity. Of course, this regime is needed to be further optimized and becomes powerful enough to offer more support to clinical diagnosis and treatment of ectopic third molar in the clinic.

## Conclusion

In conclusion, ectopic mandibular third molar is a rare clinical entity with non-specific presentations. Its location in mandible associates significantly with its clinical presentations and affects the selection of surgical approach. The benefits, potential risks and complications should be weighted carefully when surgeons manage this rare situation. Our definition and classification for this rare disease might be beneficial to aid its diagnosis and treatment.
